# Orofacial assessment as digital path for forensic and legal evidence record

**DOI:** 10.1093/fsr/owae006

**Published:** 2024-02-01

**Authors:** Tiago Nunes, Rita Ribeiro, Pedro A Almiro, Rebeca Fontes, Ricardo Machado, João Abreu, Ana Corte-Real

**Affiliations:** Faculty of Medicine, University of Coimbra, Coimbra, Portugal; Faculty of Medicine, University of Coimbra, Coimbra, Portugal; Center for Research in Psychology, Autonomous University of Lisbon, Lisbon, Portugal; Faculty of Medicine, University of Coimbra, Coimbra, Portugal; Faculty of Medicine, University of Coimbra, Coimbra, Portugal; Clinical and Academic Centre of Coimbra, Coimbra, Portugal; Faculty of Medicine, University of Coimbra, Coimbra, Portugal; Laboratory of Forensic Dentistry, Faculty of Medicine, University of Coimbra, Coimbra, Portugal

**Keywords:** human identification, forensic dentistry, forensic pathology, legal medicine digital technology, 3D image

## Abstract

In forensic scenarios, such as armed conflicts or mass disasters, the oral cavity can be a valuable source of identification information relevant to legal issues. In many European Union countries, it is mandatory to register dental records for identification purposes. A pilot and quasi-experimental study was performed. The study aims to analyze two methodologies, photography and wireless intraoral (IO) laser scanner, in the scope of the orofacial record in forensic pathology, highlighting their impact on human identification. The IO scanner i700 (Medit, Lusobionic, Portugal) and Canon 5D-Full Frame equipment were used to record the individual status, living patients (*n* = 5), and forensic cases (*n* = 5). IO and extraoral anatomical structures were recorded following six parameters: time, mineralized and soft detail, communication, extra devices, and distortion. The statistical analysis was performed in accordance with a scoring system and Mann–Whitney (*P* < 0.05) analysis. The photography method recorded extraoral data for all samples (score range between 15 and 23). The time elapsed to complete an IO scan in forensic cases was shorter than with photography, without requiring additional sources of light or mirror devices. Living patients and corpses identified statistically significant differences. It can be concluded that laser scanners are a valuable tool in the field of forensic pathology and can be used to record and analyze anatomic-morphological data for identification purposes accurately.

**Key points:**

- Human identification engages in orofacial details records.
- Photographic and laser scans record intraoral and extraoral anatomic structures.
- Forensic cases assessed by intraoral scanner technology are accurate and less time-consuming, optimizing the orofacial data for identification.

## Introduction

Human identification is a current and emerging trend in the medico-legal field. It is grounded on the comparison of antemortem (AM) or missing data and postmortem data or undocumented findings [1].

In the human identification process, fingerprint comparison systems and genotyping are the gold-standing methods because of their accuracy (over 99%) [2]. Both methods require a trustable baseline and preserved material [2]. In extreme forensic conditions, fingerprint analysis becomes unreliable. The dental procedure becomes elective for morphological analysis and genotype profile sampling [3, 4].

AM data are accessed from clinical dental records following the Data Protection Act 2018, which demands the preservation of dental records. Clinical dental records are stored as a civil database, including radiographies, photographs, dental charting, and dental models [4, 5]. These records include distinctive characteristics such as missing teeth, dental implants, prostheses, dental restorations (fillings), presence of tooth crowding, or unusual arrangements of teeth in a dental arch.

Clinical dental data records are either digital or handwritten. In contemporary dental clinical practice, clinical data can be recorded digitally trough computer-aided imaging. The resulting data files allow dental planning through computer-aided design and, ultimately, on the production of anatomical structures through computer-aided manufacturing [6]. Digitizing devices include cone-beam computed tomography (CBCT) and intraoral scanners (IOS). IOS provide a 3D record of the dental arches with a significant decrease in time, stress, and discomfort for the patient [7] when compared with the conventional dental cast impression method [5]. Furthermore, it allows easy and quick access to patient information, reduces storage space, and facilitates communication between peers.

IOS can be categorized as contact and laser [7, 8]. Contact IOS explore an object's surface through a probe with a hard-steel or sapphire tip, and a series of internal sensors determine the spatial positions of the probe. It is used in inspection and quality control systems because of several well-documented studies on its calibration process and measurement uncertainties. On the other hand, the laser IOS projects a light source. It detects its return, capturing the object’s geometry by triangulation and capturing the dental arches more rapidly than the contact IOS [8]. The object’s geometry is generated by the scanning software that processes the captured images to generate point clouds. These point clouds are then triangulated by the same software, creating a 3D surface model (mesh), producing a “virtual” alternative to traditional plaster models [3, 6, 7].

The accuracy of these scanners in recording and analyzing anatomic morphology is well established [3, 9–11]. The detailed and reliable reproductions of intraoral (IO) and extraoral (EO) anatomical structures (such as teeth, nose, lips, palate wrinkles, and bridles) can be beneficial in clinical and forensic experimental settings [3, 6, 9, 10]. A complete IOS 3D model can include other stationary structures besides teeth, allowing the association of high-reliability methods such as rugoscopy, geometric analysis, and radiological comparison [5].

Recent data from the International Organization for Migration have shed light on the global impact of personal data requirements. According to the 2022 report [12], 3.60% of the world's population, equivalent to 281 million people, are migrants in 2020. This underscores the importance of a global shared space with standardized recording formats, facilitating effective communication of personal data, analysis, and comparison [13, 14].

The primary purpose of this study is to analyze two methodologies, photography and laser scanner, in the scope of the orofacial record in forensic pathology, highlighting their impact on human identification on real forensic scenarios.

## Material and methods

### Sample selection

Oro-pathological examinations, into a standard autopsy protocol, were performed at the National Institute of Legal Medicine and Forensic Science (INMLCF, IP), Central Branch, Portugal, between June and December 2022, and encompassed forensic cases of individuals over 18 years of age, following Portuguese protocol, present in the Portuguese’s Record of Non-Donors (RENNDA). The exclusion criteria were (i) fragmented and/or (ii) undocumented corpses, (iii) advanced decomposition and (iv) edentulous status. Dental examinations of living individuals were performed at the Laboratory of Forensic Dentistry (LFD), Faculty of Medicine, University of Coimbra, Portugal, between June and December 2022, in adult patients. The exclusion criteria were (i) edentulous status and (ii) removable dental prosthesis. Written informed consent was obtained from all participants.

The study was conducted according to the Declaration of Helsinki and approved by the Ethics Committee CE12/2022 of the INMLCF, IP and CE-023/2027 of the LFD.

### Study design

A pilot and quasi-experimental study design was conducted to examine oral and face records acquired in forensic cases and living patients, by two methods. The data were obtained by: (i) photography and (ii) IOS methods.

The photography method used the Canon EOS 5D Mark-II (Tokyo, Japan) and Macro 100-mm lent equipment to photograph frontal and lateral EO views and both upper and lower dental arches [8, 10]. A standardized photograph status was performed, by the same two operators, one to take the photo and the other to ensure the correct placement of the accessories, lip retractors (OptraGate, Ivoclar®, Schaan, Liechtenstein), supplementary lighting (flash), and tripods (monkey tripod, Joby®) [10]. The photographs were exported to a MacBook Air M2 24 GB of RAM (Apple Inc., Cupertino, CA, USA). One photo “status” corresponded to record: frontal, laterals (left and right), upper arch, lower arch, and facial views.

**Figure 1:**
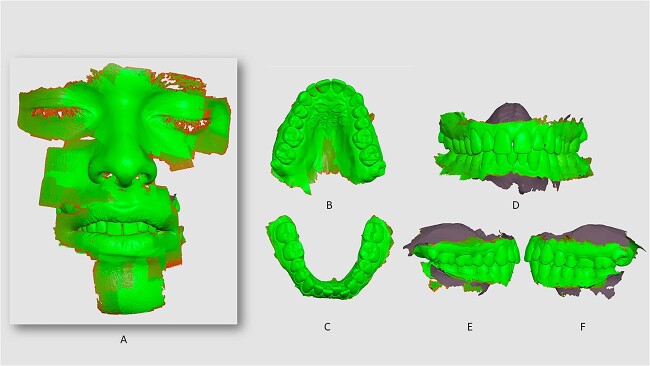
Intraoral scanner (IOS) record *status* (sample L3), under the resolution software scale (red to green) in which green corresponds to a better performance of the scanning process: facial scan (A), upper arch (B), lower arch (C), frontal (D), lateral right (E) and lateral left (F) views.

The IOS method used the i700 wireless IO laser scanner (Medit, Lusobionic, Portugal), and Medit Scan for Clinics software to collect EO and IO scanning details [13]. A standardized scan was performed by the same operator. IOS tip was positioned as close as possible to the teeth, starting on the occlusal surface of the left rearmost teeth, continuing through the palatal/lingual surfaces, of the upper and lower arches, and the environmental soft tissues. EO scanner was performed in reference to the smile design and anatomic details in circumference until the record of facial structures of the face. The 3D-coloured images were exported to the Medit software on a MacBook Air M2 24 GB of RAM (Apple Inc). One 3D model “status” corresponded to a 3D model, allowing to record: frontal, laterals (left and right), upper arch, lower arch, and facial views (Figure 1).

The study focused on the lower one-third of the face, between “sub nasale” (Sn) and “menton” (Me) soft tissue points, following Arnett’s study [11]. Both techniques following the parameters recorded EO and IO areas: time elapsed (seconds), mineralized details (teeth identification), soft details (nose, lips, palatal rugae, and bridles identification), communication (bi- or tridimensional record), extra devices (mirror, auxiliary light, and fixed support), and distortion (significant and requires edition procedures or insignificant). The face details were recorded by both techniques.

Mineralized and soft structures were analyzed following the partial or total record of anatomical details. The whole mineralized tissues correspond to the teeth of both dental arches, as complete IO soft tissues correspond to the palate rugae and IO bridles.

### Data analysis

The research team, with 15 years of experience in medico-legal expertise and anatomy education [14, 15], analyzed the data records’ consistency by (i) data cross-validation, (ii) referenciation databases standards, (iii) engaging experts in the field, and (iv) implementation validation rules.

The parameters of mineralized details, soft tissue details, communication, extra devices, and distortion were evaluated for both IO and EO views of living individuals (*n* = 30) and dead individuals (*n* = 30) using photography and IOS methods. The evaluation was based on a scoring system following a Likert scale (0–4), in which higher scores were given for better performance in each parameter [15]. For the mineralized and soft details parameters, a score of 4 points was given if all elements were visible in both jaws, 3 points if all features were visible in one jaw and partially visible in the other jaw, 2 points if details were partially visible in both jaws, 1 point if details were partially visible in one jaw and not visible in the other jaw, and 0 point if no elements were visible in both jaws. The C score was based on the type and format of data included in the record. A score of 4 points was given if the document had both 3D and 2D data, 3 points for only 3D data, 2 points for only 2D data with colour information, 1 point for only 2D data without colour information, and 0 point for unformatted data. The Ed scale was based on the number and type of devices used. A score of 4 points was given if no extra devices were used, 3 points if mouth retractors were used, 2 points if additional lighting was used, 1 point if both mouth retractors and additional lighting were used, and 0 point if mouth retractors, extra lighting, and a tripod were used.

Distortion was scored with 4 points if the records had no distortion, 3 points if the documents needed a dimensional scale for some record details to be assessed, 2 points if a scale was required for some of the details to be evaluated, 1 point if the records showed partial distortion, and 0 points if the records were entirely distorted.

A statistical study was performed by descriptive analysis, and the Mann–Whitney nonparametric tests were used to compare groups [16]. The level of statistical significance was set at least *P* < 0.05.

## Results

Five oro-pathological examinations (D1–D5) and five dental examinations (L1–L5) were selected between 150 examinations to fill the sample criteria. The sample included Portuguese individuals from 19 to 65 years old, with a mean age of 33.9 ± 14.7, and the majority were men (80%).

The i700 scanner was able to record IO data for all samples (*n* = 10). All individual scores were summed up and presented in Table 1.

**Table 1:** Descriptive statistics for i700 scanner (*n* = 10).

Parameters	Mean±SD	Minimum		Maximum	
		Life	Death	Life	Death
IO					
Mineralized	4.00±0.00	4	4	4	4
Soft	4.00±0.00	4	4	4	4
Communication	4.99±0.00	4	4	4	4
Extra devices	3.50±0.53	4	3	4	3
Distortion	4.00±0.00	4	4	4	4
EO					
Soft	2.80±1.32	1	4	2	4
Communication	4.00±0.00	4	4	4	4
Extra devices	4.00±0.00	4	4	4	4
Distortion	3.40±1.27	1	4	4	4

IO: intraoral; EO: extraoral.

**Figure 2:**
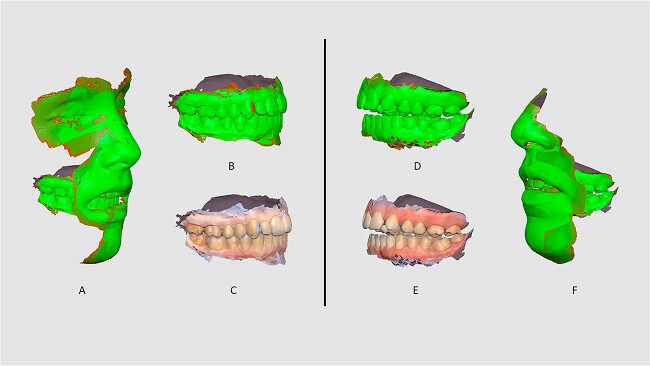
Intraoral scanner (IOS) “status” analysis (sample D5—A–C; and sample L1—D–F).

The scanner performed with the lowest results in EO view in living patients. EO data corresponded to the Minimum 1 score and Maximum 2 scores for the soft tissue record parameter and the Minimum 1 score and Maximum 4 scores for the distortion parameter (Table 1; Figure 2). The scanner performed with high scores in all other parameters, either in living patients or corpses.

The photography method was able to record EO data for all samples (*n* = 10). All individual scores were summed up and presented in Table 2. Different devices, mouth retractors, and additional lighting were used (1 score). IO mineralized data were partially recorded in the corpses (between 2 and 3 scores) and highlighted the lower score of IO soft details (between 0 and 2 scores).

**Table 2:** Descriptive statistics for photography (*n* = 10).

Parameters	Mean±SD	Minimum		Maximum	
		Life	Death	Life	Death
IO					
Mineralized	3.10±0.99	4	2	4	3
Soft	1.70±0.95	2	0	3	2
Communication	2.00±0.00	2	2	2	2
Extra devices	1.00±0.00	1	1	1	1
Distortion	2.30±0.82	3	1	3	2
EO					
Soft	4.00±0.00	4	4	4	4
Communication	2.00±0.00	2	2	2	2
Extra devices	1.00±0.00	1	1	1	1
Distortion	2.70±0.48	3	2	3	3

IO: intraoral; EO: extraoral.

**Figure 3:**
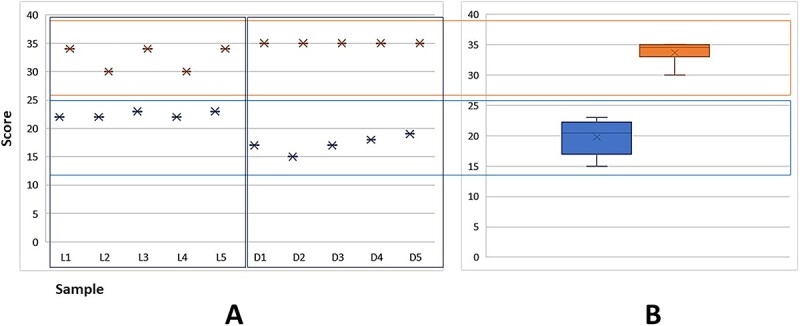
(A) The corresponds to the summed scores for live patients (L1–L5) and corpses (D1–D5) (*n* = 10). (B) A boxplot graphic with a score between photographic (lower rectangle) and intraoral scanner (IOS) (upper rectangle) (*n* = 10).

The descriptive analysis included the summed scores of each parameter for all samples (Figure 3). The lower values corresponded to photographic methods on corpses (Score 15). All photographic scores were between 15 and 23, the highest scores in living patients.

The higher values were found on IOS methods (Table 3). The lowest score amongst IOS methods was 30 in living patients, and the highest score was 35 in corpses.

**Table 3:** Descriptive statistics for time (second) parameter (*N* = 40).

Parameters	*n*	Mean±SD	Minimum	Maximum
Photography IO	10	601.30±314.07	300	907
IOS IO	10	754.00±34.94	710	800
Photography EO	10	195.70±112.72	86	308
IOS EO	10	104.00±21.06	75	130

IO: intraoral; IOS: intraoral scanner; EO: extraoral.

### Living patients and forensic cases comparison

The analysis by Mann–Whitney test between living patients and corpses identified differences statistically significant in IOS method performance for IO extra devices (*P* = 0.003) and EO soft tissues (*P* = 0.005).

The analysis by Mann–Whitney test between living patients and corpses identified differences statistically significant in photographic method performance for IO mineralized (*P* = 0.004), IO soft tissues (*P* = 0.016), and IO distortion (*P* = 0.005).

## Discussion

The present study highlighted wireless IOS in oro-pathological examinations for medico-legal and forensic purposes in real forensic human identification scenarios, challenging the traditional use of photographs as a routine complementary diagnostic examination 3D models in digital format, instead of 2D information obtained from photography, mean more significant information in the data-evidence chain, in immediate sharing between field professionals and the institutions that host the information for data comparison. A digital archive of personal data, for judicial purposes, using the same methodology as clinical or AM data archive [5], allows faithful comparison and virtual presentation in court [3, 10] and between professionals, as a technology “based in identity” [17].

This study’s findings impact one of the most challenging situations of the current migration crisis, which is the need to obtain personal data, especially from undocumented people [14–21]. Digital storage of big data, as a tool for clinical, forensic, and social professionals, reliably manages personal data [12, 14]. IOS technology applied to a complete record of facial and oral data should be used as the digital path for recording forensic and legal evidence of mineralized and soft tissues in this area of excellence in human identification.

The present study analyzed six parameters by comparing the IOS and photographic methods.

Compared with the photographic method, recording EO data by IOS, without needing extra devices, has the highest performance in cadavers. The IOS technique has static structures as its reference, so in the living, remaining in the same position can be a problem, particularly at the level of the middle floor of the face where the light from the end of the equipment infers with “eyes closed”.

The acquisition of EO data by IOS, without extra devices, has better performance in cadavers, namely, soft tissue records (*P* = 0.005) and shorter duration (maximum 130 *versus* 308 s, for photographic method). The IOS technique uses static structures as its reference, so remaining in the same position can be a problem for the living, particularly at the level of the middle floor of the face, where the light coming from the end of the equipment infers the occlusion position of the eyelids.

The acquisition of IO data by IOS, without extra devices (*P* = 0.003), was the election for all samples, requiring less time (duration) than the photographic procedure (maximum 800 *versus* 907 s). It has better performance in cadavers without the need for an additional linear incision on the face, as required by Putrino et al. [5]. During oral pathology examination, the corpse rigidity limits mouth opening, preventing the photography method’s ability to fully record all the structures without the necessity of incisions that will damage the facial integrity of the corpse [5].

Comparing living patients and corpses, distortion was not a concern when using IOS. However, it can be an issue when using photographic procedures (*P* = 0.005), particularly in the molar region following Bastos et al. [10] highlighting IOS as a reality of modern dentistry within clinical and forensic perspectives.

For forensic purposes, the morphology of the dental crown is complemented by the analysis of the entire dental arch, including the study of the palatal wrinkles, following the systematic review by Vilborn and Bernitz [9]. The sensitivity and specificity of oral scan data were confirmed in Simon’s study, in which geometric comparisons of oral scans between monozygotic and dizygotic twins displayed that identical individuals were correctly identified with high sensitivity (91.2%) and specificity (97.8%) [20]. IOS technology appears to be the highest precision method in both complete- and partial-scan data, following Bae and Woo’s studies [3]. In addition, Bastos et al. [10] highlighted the advantages of IOS technology in clinical scenarios, compared with photography procedures, overcoming camera failure positioning, poor focusing, and over- or underexposure. The possibility of manipulating the image along various axes on IO scanning is ideal for visualizing small anatomical structures and for easy storage of high-quality images for a detailed comparison and evaluation of unlimited exposition plans. In this sense, IOS should replace plaster models and IO photographic recording to precisely record all personal data, engaging the challenging of palatal rugae [20] analysis on “identity base” technology [21].

Technological advancement and the disruptive integration of new technologies are essential in the precision of details and the optimization of information analysis, as emphasized by Simon and Pacifici’s studies [20, 21]. In this sense, the present study’s presentation and use of cutting-edge technology align with the IOS trend in promoting the impact of facial structures’ anatomical and functional relationship. This digital path includes artificial intelligence in applying algorithms that validate the scanned forms and optimizes their visual reproduction [13]. The reliability of the equipment (i700) emphasizes greater precision in detecting anatomical limits, namely, the sharp edges of the incisal edge of the anterior teeth; the relationship between the same structures in different registers, namely, between the dental arches and the scan of the face in support of the lips, the relationship between the two dental arches through some occlusion points. The significant reliability, ease of handling, and reproducibility of 3D models are associated with software (Medit) in which artificial intelligence is used, revealing IOS to be a potentially accurate and robust tool with forensic applicability [3, 5].

The learning curve for using IOS can represent a bias. It may vary depending on the specific model and software and the individual’s previous experience with similar technology.

The high reliability, ease of handling, and reproducibility of 3D models make IOS a potentially accurate and robust tool with forensic applicability [3] to aid the confirmation of human identity [5].

## Conclusion

The present study demonstrated the benefits of using IOS in pathology examinations for medico-legal and forensic purposes, particularly in real forensic human identification scenarios, comparatively with traditional photographic methods. The time required to complete an IO scan in forensic cases was shorter than the time needed for a conventional photographic procedure, without requiring additional sources of light or mirror devices.

IOS, the laser scanner, was highly reliable, with high precision in detecting sharp edges and low distortion. The high reliability, ease of handling, and reproducibility of 3D models make IOS a potentially accurate and robust tool format, allowing for proper and real-time communication and comparison.
